# Lifestyle and eating habits in a business community.

**Published:** 2014-04-08

**Authors:** L Stefani, L Francini, C Petri, G Mascherini, I Scacciati, N Maffulli, G Galanti

**Affiliations:** 1 Sport s Medicine Center, Florence, Italy.; 2 Department of Musculoskeletal Disorders, Faculty of Medicine and Surgery, University of Salerno, Salerno, Italy; and Queen Mary University of London, Barts and The London School of Medicine and Dentistry, Institute of Health Sciences Education, Centre for Sports and Exercise, UK

## Abstract

**PURPOSE:**

The present study verified, using a validated questionnaire, the presence of unhealthy aspects of lifestyle and chronic degenerative conditions in a working community.

**METHODS:**

A cohort from a working community in Italy was investigated using of the INRAN (Istituto Nazionale di Ricerca per gli Alimenti e Nutrizione) questionnaire dedicated to the eating habits and Physical Activity Stages of Change.

**RESULTS:**

Most of the 93 subjects (56 females and 37 males, aged 42.0±0.7) recruited reported low levels of physical activity (70 subjects). Slightly more than 50% of the subjects undertook physical activity more than once a week, while 13% did it only once. Food intolerances were reported by 7 subjects (8%), with a high consumption of fruits, cereals and dairy products, low consumption of fish and alcohol, and meat consumption in the normal range. There was a high satisfaction in general quality of life.

**CONCLUSION:**

Questionnaire investigations play a role to identify the presence of degenerative chronic conditions in working communities. The self-reported perception of quality of life does not necessarily agree with the lifestyle habits found. Awareness of this aspect could be helpful to plan lifestyle interventions and promote healthy living habits.

## Introduction

Regular physical activity plays a positive role in the prevention of many chronic conditions, including diabetes, cardiovascular disease, colon cancer and depression [1,2,3]. Physical inactivity and sedentarism are a major cause of increased health care costs[4]. Particularly in the workplace, they can have a wide potential negative effect on production output at work. Several studies have detailed worksite health promotion program participation [5]. Questionnaires are one of the most direct methods to objectify this aspect, and are an easy method to identify sedentarism associated with poor dietary habits[6]. The present study verifies the feasibility to use a validated questionnaire as a simple tool to detect some aspects of lifestyle related to a the presence of chronic degenerative diseases in a small working community.

## METHODS

A small sample from a working community of a pharmaceutical company in Tuscany-Italy composed of 93 subjects(56 male and 37 female, average age 41±9.4 years) was investigated The adhesion to the study was voluntary, and formal oral and written consent were obtained. The lifestyle investigation was conducted using the INRAN (Istituto Nazionale di Ricerca per gli Alimenti e Nutrizione) questionnaire for eating habits and Physical Activity Stages of Change for tendency to exercise questionnaire[6]. The questionnaire is composed of three different parts.

One part is dedicated to study physical activity (PA) habits, the second describes the eating habits (EH) of the study population, and the third evaluates the self perception of quality of life (QoL).

All the three parts of the questionnaire were analyzed on the basis of the self-reported score. Each score obtained was interpreted from the range achieved. The amount of the programmed weekly physical activity (PA) performed was quantified as “never”, “once per week”, “more than once per week”. The first and the second level corresponded to sedentarism, the third to a larger range, including low and moderate PA level.

For the QoL global investigation, the evaluation was based on the analysis of some parameters of the “self lifestyle perception”. Among them, the principal questions were addressed to investigate the amount of the free time available, the perception of the subject’s own cognitive abilities, quantity of time dedicated to work or study, time for socialization activities, time dedicated to physical activity, time for recreational activity. Four different intervals were considered: > 40 excellent, > 31 < 40 Fair, > 25 < 31 Good, >11 < 25 Unsatisfactory, < 11 Absent. The relevant score was used for each survey.

Regarding the EH, the evaluation of the data was derived from the answers directly expressed in the questionnaire.

## Statistical analysis

All the data are expressed as percentages. Analysis of the data was undertaken using Microsoft Office Excel (Microsoft Coporation, USA). The correlation was possible by the Spearman’s correlation coefficient. The scale of magnitude for correlation was trivial 0<r<0.1, small 0.1<r<0.3, moderate 0.3<r<0.5, large 0.5<r<0.7, very large 0.7<r<0.9, nearly 0.9<r<1, perfect r=1 (biblio Hopkins WG. A scale of magnitudes for effect statistics. 2009. Dec, Available at:http://www.sportsci.org/resource/stats/index.html.)

## Results:

### General data

The sample was composed of 93 subjects (56 females and 37 males) aged 41.0±9.4, of whom 11 subjects (12%) were smokers. Chronic degenerative conditions were reported by 33 subjects corresponding to 35% of the group, with a prevalence of 67% (22 subjects) in female and 33% (11 subjects) in male. The rest of subjects (65% of whole group) reported to be healthy. Within the group reporting chronic degenerative conditions, cancer was reported in 6 subjects (21%), hypertension in 5 subjects (17.5%), coronary artery disease in 1 subject (3.5%), autoimmune disease in 5 subjects (17%), hypothyroidism in 2 subjects (7%), hyperthyroidism in 1 subject (3.5%), and multiple sclerosis in 1 subject (3%). Only 6 subjects (21% of the subjects) reported otherwise to be not fully healthy, and therefore included in a separate group. There was a high prevalence of overweight subjects, more in males (BMI > 25 in 17 subjects, 65%) than in females (BMI > 25 in 9 subjects, 35%). In males, only 22% had a BMI < 25, while 18% were overweight. In females, 48% had a BMI < 25, and 12 % were overweight.

### Physical activity

The population studied reported to adhere to a correct lifestyle; 75% of the whole population was active, while only 25% were sedentary, without differences between the genders:. In 51% of the whole group, PA was regularly practiced more than once a week, while 13% undertook PA only once a week (Fig 1). Programmed physical activity was more often reported in males (62% male vs 38% female), as well as spontaneous physical activity (65% male vs 41%) female. BMI was < 25 in the subjects who undertook PA at least once a weak. The percentage of the risk factors (BMI > 25 and smoking) within the different PA levels showed a trend to have a lower BMI in subjects undertaking PA more than once per week. In the same group, the incorrect lifestyle habits, including smoking, were however maintained.

There was evidence of a statistically significant negative association between BMI >25 values and Qol (r=−0.88) (*p<0.0001*).

### Eating Habits and quality of life perception

Food intolerances were reported by 8% of the subjects involved in the present investigation. There was a high consumption of fruits, cereals and dairy products, with low consumption of fish and alcohol. Meat consumption was within normal limits, and in agreement with the literature and IRAN guidelines (7).

### Quality of life perception

The perception of QoL was high: in 70% of the community, with a range from 60% to 90%.(Fig 2, Fig 3).

## Conclusions

There is a definite relationship between health climate at work and well being[7]. The analysis of lifestyle in workers’ communities is a new aspect in promoting health and in engaging the participants in the self-assessment of their own habits associated to well defined risks factors. Sedentarism is often present in these communities[8]. Dedicated questionnaires are limited tools to define an individual’s behavior, but they are helpful and sensitive instruments for a first investigation of large cohorts[9]. We acknowledge that this is a pilot study: the data obtained are in agreement with the current literature, where the prevalence of the main metabolic diseases is higher in sedentary subjects. The group investigated is relatively poorly active, and the percentage of the subject with chronic diseases is relatively high, especially when considering cancer, and in agreement with the current literature [10]. A statistically significant association between BMI and PA was confirmed. This supports the importance to take care of one’s own lifestyle in terms of spontaneous and programmed daily physical activity whose intensity should be within low to medium to reach a therapeutic effect. On the contrary, a high level of quality of life perception was reported in both males and females, demonstrating as self perception can represent a confounding parameter in case of degenerative chronic diseases screening.

The present investigation highlights the possibility to obtain using a questionnaire much information on several aspects of physical activity, eating habits and prevalence of risk factors. In this way, an initial picture of a given population can be obtained and interventions aimed at changing lifestyle habits can be planned and therefore monitored. More information can be also obtained using accelerometry, considering also that accelerometry data are often well integrated with the questionnaire data in the interpretation of lifestyle habits [11].
